# Immunoreactivities of androgen receptor, estrogen receptors, p450arom, p450c17 proteins in wild ground squirrels ovaries during the nonbreeding and breeding seasons

**DOI:** 10.1186/1757-2215-5-26

**Published:** 2012-09-25

**Authors:** Xiaonan Li, Haolin Zhang, Xia Sheng, Ben Li, Jiao Zhou, Meiyu Xu, Qiang Weng, Gen Watanabe, Kazuyoshi Taya

**Affiliations:** 1 College of Biological Science and Technology, Beijing Forestry University, Beijing, 100083, China; 2 Laboratory of Veterinary Physiology, Department of Veterinary Medicine, Faculty of Agriculture, Tokyo University of Agriculture and Technology, Tokyo, 183-8509, Japan; 3 Department of Basic Science, United Graduate School of Veterinary Sciences, Gifu University, Gifu, 501-1193, Japan; 4 Laboratory of Animal Physiology, College of Biological Science and Technology, Beijing Forestry University, Beijing, 100083, People’s Republic of China

**Keywords:** Androgen receptor, Ovary, P450c17, P450arom, Wild ground squirrels

## Abstract

The aim of this study was to elucidate the regulatory role of androgen in the follicular development of wild female ground squirrels. Immunohistochemical staining of FSHR, LHR, P450c17, P450arom, androgen receptor (AR), estrogen receptors (ERa and ERb) were executed in ovaries of female ground squirrels from both breeding and nonbreeding seasons. In addition, total ovarian proteins were extracted from the ovaries of squirrels from breeding and nonbreeding seasons, and Western blot analysis were performed in order to probe for FSHR, LHR, P450c17, P450arom, AR, ERa and ERb. The results of immunohistochemical staining and Western blotting of P450c17 showed that there was no significant difference between the breeding and nonbreeding seasons. It was found that granulosa cells expressed P450arom during the breeding season. In contrast, there was no positive staining of P450arom in the nonbreeding season. There was no significant difference in immunoreactivity of AR between the breeding and nonbreeding seasons. However, the immunoreactivities of ERa and ERb were both significantly reduced in the nonbreeding season compared to the breeding season. The positive stains of FSHR and LHR were found in the granulosa cells and theca cells of the ovaries of the breeding and nonbreeding seasons. In addition, the Western blotting results of FSHR and LHR showed a significant reduction in the nonbreeding season compared with the breeding season. These findings suggested that androgen might be predominantly converted into estrogen in order to regulate the follicular development via binding of estrogen receptors during the breeding season, whereas androgen might predominantly directly bind androgen receptor to regulate the follicular development during the nonbreeding season in the ovaries of wild female ground squirrels.

## Introduction

The major stages of ovarian folliculogenesis were formation of the primordial follicle; recruitment into the growing pool to form a primary, secondary, and tertiary follicle; and lastly ovulation and subsequent formation of a corpus luteum (CL) [1]. These physiological progressions were under the regulation of hypothalamic-pituitary-gonad (HPG) axis [2]. GnRH from the hypothalamus stimulated the anterior pituitary to secrete follicle-stimulating hormone (FSH) and luteinizing hormone (LH), which acted on the ovary to promote folliculogenesis and the concomitant synthesis of estradiol [3]. Following follicular recruitment was the gonadotropin-independent stage, a state in which preantral follicular development did not require stimulation by the pituitary gonadotropins [1]. Subsequent to this was the gonadotropin-dependent stage, which was when preantral follicles grew to anrtral follicles. Thereafter, secretion of FSH by the pituitary promoted further granulosa cell proliferation and survival. Ovulation of the dominant follicle occurred in response to a rise in the other pituitary gonadotropin, LH [1]. Under the regulation of LH and FSH, theca cells and granulosa cells produced androgen and estrogen, respectively. Androgen and estrogen biosynthesis was catalyzed by a member of the cytochrome P450 surperfamily, namely cytochrome P450 17a-Hydroxylase/c17-20Lyase cytochrome P450 (P450c17, the production of *CYP17A1* gene) and aromatase cytochrome P450 (P450arom, the production of *CYP19* gene), respectively [4]. The expression of P450c17 and P450arom in the ovary had been reported in many species, inclu-ding rat [5], bovine [6,7], human [8], goat [7,9], Japanese Shiba goat [10] and mice [11].

In the rodent ovary, estrogen was primarily produced by preovulatory follicles under the influence of FSH [12]. The well-documented endocrine actions of estrogen in the ovary were critical to reproduction, and signaled via two nuclear estrogen receptors, ERa and ERb [13]. Androgen mediated their action primarily via AR, a member of the nuclear receptor superfamily encoded by an X chromosomal gene [14]. Androgen and AR had defining roles in male reproductive development and function [15]. In contrast, little was known about the actions of androgen and AR in female reproduction, although AR expression in growing follicles had been described [16]. Previous research suggested that AR was most abundant in the granulosa cells of rat ovaries and the expression of AR and its mRNA were developmentally regulated, being down-regulated during FSH-stimulated preovulatory follicular development [17].

The wild female ground squirrel (*Citellus dauricus* Brandt) was a typical seasonal breeder, with a breeding season from April to May. From June to March, however, was the time of hibernation, or the nonbreeding season, in which the wild ground squirrel went through a long period of sexual dormancy [18-20]. The wild female ground squirrel provided us with a useful model to study the role of androgen in follicular development during the breeding and nonbreeding seasons. Previously, our evidence had implicated that inhibin and activin might play an essential role in the regulation of seasonal folliculogenesis in the wild ground squirrel [21]. The aim of the present study was to investigate immunoreactivities of FSHR, LHR, AR, ERs, P450arom, P450c17 proteins during the breeding and nonbreeding seasons, and to elucidate the regulation role of androgen on the follicular development in wild female ground squirrels.

## Materials and methods

### Animals

Twenty wild female ground squirrels thought to be adults based on their body weight (242-412g) were captured in April or May (breeding season, n = 10) and August or September (nonbreeding season, n = 10) from 2010 to 2011 in Hebei Province, P.R. China. All procedures involving animals were carried out in accordance with the Policy on the Care and Use of Animals, approved by the Ethics Committee, Beijing Forestry University, and approved by the Department of Agriculture of Hebei Province, P.R. China (JNZF11/2007). The animals were euthanized by decapitation before tissue removal within 24h of capture and the ovarian tissues were obtained. One part of the samples were immediately fixed in 4% paraformaldehyde in 0.05M PBS (PH 7.4) for histological and immunohistochemical observations; the other part of the samples were immediately frozen in liquid nitrogen and stored at -80°C for Western blotting.

### Histology

Ovarian samples were dehydrated in an ethanol series and embedded in paraffin wax. Serial sections (4-6μm) were mounted on slides coated with poly-L-lysine (Sigma, St. Louis, MO, USA), and stained with hematoxylin-eosin (HE) for observation of general histology. Follicles in the differential developmental stages were evaluated histologically using an Olympus photomicroscope with 10 × objective lens. Meanwhile, the statistical number of follicles in different developmental stages was examined. Primary follicles showed a single layer of cuboidal granulosa cells. Secondary follicles possessed more than one layer of granulosa cells with no visible antrum. Antral follicles possessed one or two small areas of follicular fluid (antrum). Dominant follicles had a rim of cumulus cells surrounding the oocyte. All follicles were estimated by exact counts determined from 4-6 μm paraffin sections from 10 different ovarian samples of wild female ground squirrels. Image analysis software (Jandel Scientific Sigma Scan; Jandel Scientific, Montgomeryville, PA, USA) was used for processing measurements.

### Immunohistochemistry

The serial sections of ovaries were incubated with 10% normal goat serum to reduce background staining caused by the secondary antibody. The sections were then incubated with primary antibody (rabbit polyclonal antibody) of P450c17 [22], P450arom [23], FSHR (Santa Cruz Biotechnology Inc., Santa Cruz, CA, USA), LHR (Santa Cruz Biotechnology), AR (Santa Cruz Biotech-nology), ERa (Santa Cruz Biotechnology) and ERb (Santa Cruz Biotechnology) for 12h at room temperature. The sections were then incubated with a second antibody, goat anti-rabbit lgG conjugated with biotin and pero-xidase with avidin, using a rabbit ExtrAvidin staining kit (Sigma, St. Louis, MO, USA). This was followed by visualizing with 30mg 3, 3-diaminobenzidine (Wako, Tokyo, Japan) solution in 150ml of 0.05mol Tris-HCl buffer (PH 7.6) and 30μl H_2_O_2_. Finally, the reacted sections were counterstained with hematoxylin solution (Merck, Tokyo, Japan). The control sections were treated with normal rabbit serum (Sigma, St. Louis, MO, USA) instead of the primary antibody.

### Western blotting

Ovarian tissue was diced into small pieces using a clean razor blade. The tissue was homogenized in a homogenizer containing 300 μl of 10mg/ml PMSF stock and incubated on ice for 30 min while maintaining the temperature at 4°C throughout all the procedures. Homogenates were centrifuged at 12,000 × g for 10 min at 4°C. Protein extracts (25μg) were mixed with an equal volume of 2× Laemmli sample buffer. Equal amounts of each sample were loaded and run on a 12% SDS-PAGE gel at 18V/cm and transferred to nitrocellulose membranes using a wet transblotting apparatus (Bio-Rad, Richmond, CA, USA). The membranes were blocked in 3% BSA for 1h at room temperature. Primary incubation of the membranes was carried out using a 1:500 dilution of rabbit anti-P450c17 antibody and rabbit anti-P450arom antibody, a 1:200 dilution of rabbit anti-FSHR, anti-LHR, anti-AR, anti-ERa and anti-ERb antibody for 60 min. Finally, the membrane was colored with 25 mg 3, 3-diaminobenzidine (Wako, Tokyo, Japan) solution in 25 μl TBS-T buffer (0.02 M Tris, 0.137 M NaCl and 0.1% Tween-20, PH 7.6) plus 3μl H_2_O_2_. b-actin was used for the endogenous control. Densitometric analysis of signals was quantified using Quantity One software (Version 4.5, Bio-Rad Laboratories, Inc., Hercules, CA, USA) and the optical density was calculated.

### Statistical analysis

Mean values (± SD) were calculated and analyzed using one-way ANOVA. Duncan’s multiple comparison test was used for detection of significant differences using the SPSS computer package.

## Results

### Histology

**Figure 1:**
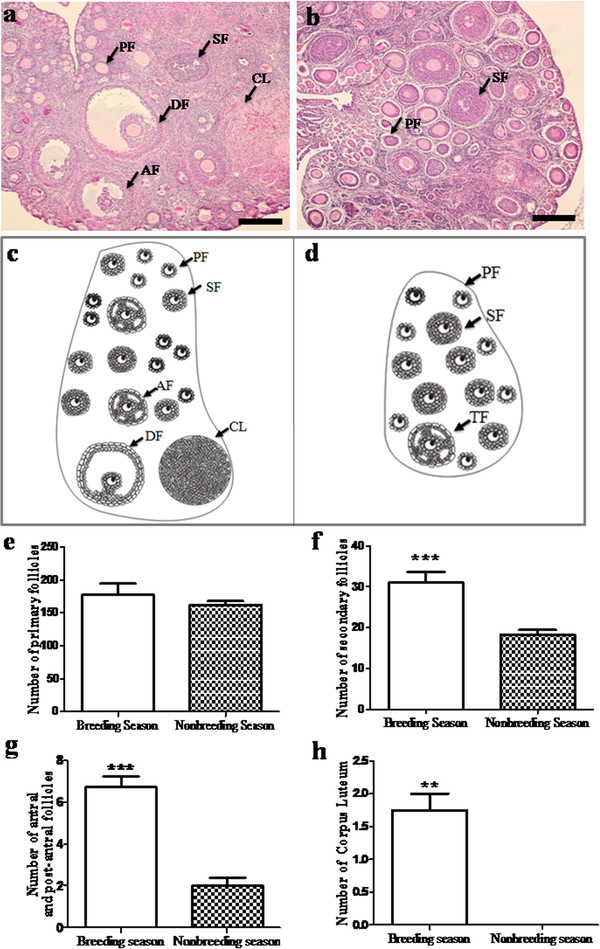
**Seasonal changes in the ovary of wild female ground squirrels.a**: The histological observation and diagram of the ovary in breeding season. **b**: The histological observation and diagram of the ovary in nonbreeding season. **c**: Histological ovarian pattern diagram of the wild ground squirrels during the breeding season. **d**: Histological ovarian pattern diagram of the wild ground squirrels during the nonbreeding season. **e**: Seasonal change of the number of primary follicles. **f**: Seasonal change of the number of secondary follicles. **g**: Seasonal change of the number of antral, post-antral follicles. **h**: Seasonal change of the number of corpus luteum. PF, Primary Follicle; SF, Secondary Follicle; AF, Antral Follicle; DF, Dominant Follicle; CL, Corpus Luteum. Data are shown as the mean ± SEM. *: p < 0.05, **: p < 0.01, ***: p < 0.001. Bars = 200μm.

The ovarian histology of wild ground squirrels showed significant seasonal changes in appearance of the follicular composition between the breeding and nonbreeding seasons (Figure 1). Follicles were observed at every different stage of the follicular development in the ovaries of the breeding season, including primary follicle, secondary follicle, antral follicle, dominant follicle and corpus luteum (Figure 1a). Only primary follicles and secondary follicles were found in the ovaries of the nonbreeding season (Figure 1b). Histological pattern diagrams of ovaries were shown in Figure 1c (the breeding season) and Figure 1d (the nonbreeding season). Except for the primary follicle, the number of secondary follicles, antral follicle, post-antral follicle and corpus luteum displayed a significant decrease in the nonbreeding season when compared with the follicle number in the ovaries of the breeding season (Figure 1)e, f, g and h.

### Immunohistochemistry

**Figure 2:**
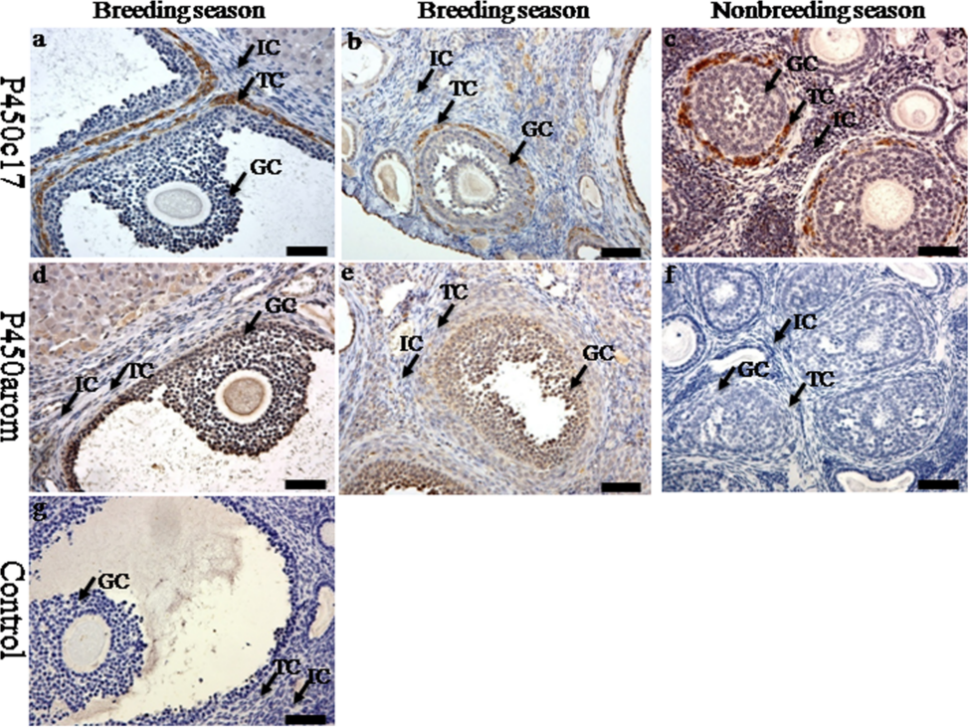
**The immunolocalization of P450c17 and P450arom in the ovary of wild female ground squirrels.** The immunolocalization of P450c17 (**a**, **b**, **c**) and P450arom (**d**, **e**, **f**) in the ovary of wild female ground squirrels during the breeding and nonbreediang seasons. The breeding season divided into two stages, the large follicle stage (**a**, **d**) and the small follicle (**b**, **e**). **g**, negative control. IC, interstitial cell; TC, theca cell; GC, granulosa cell. Bars =50μm.

**Figure 3:**
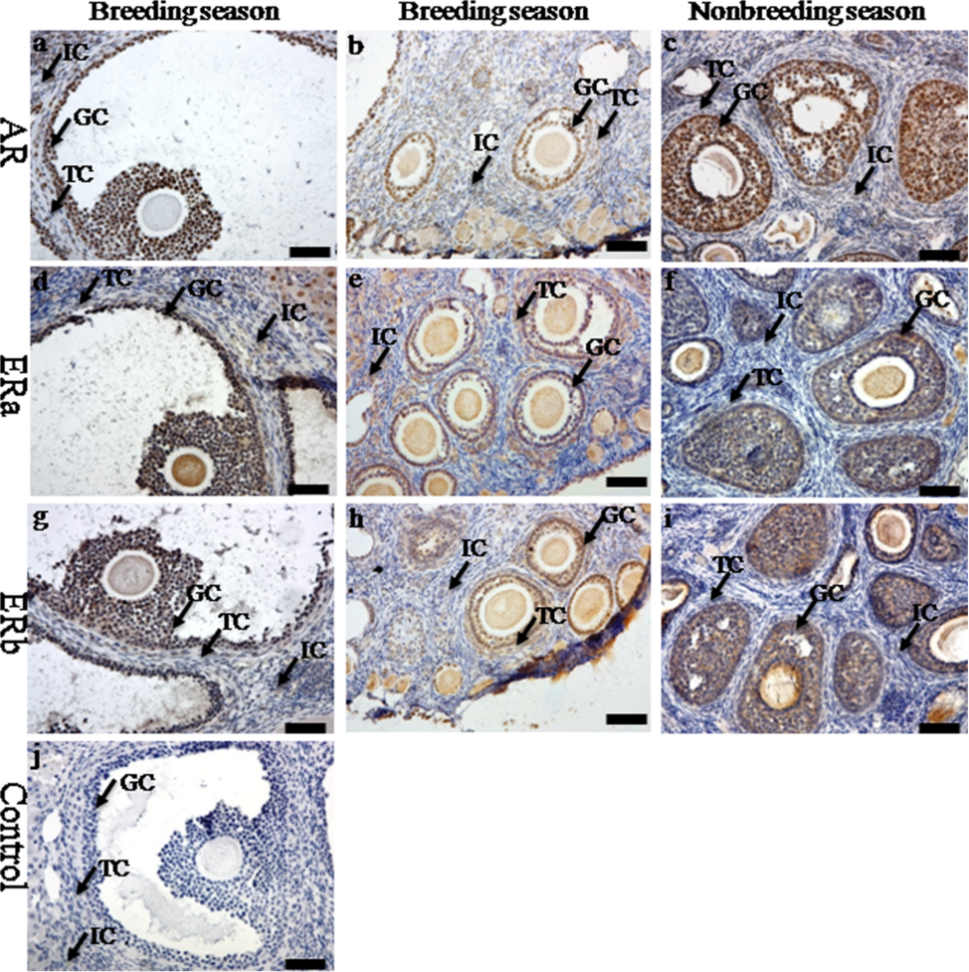
**The immunolocalization of AR and ERs in the ovary of wild female ground squirrels.** The immunolocalization of androgen receptor (**a**, **b**, **c**), ERa (**d**, **e**, **f**) and ERb (**g**, **h**, **i**) in the ovary of wild female ground squirrels during the breeding and nonbreediang seasons. The breeding season divided into two stages, the large follicle stage (**a**, **d**, **g**) and the small follicle (**b**, **e**, **h**). **j**, negative control. IC, interstitial cell; TC, theca cell; GC, granulosa cell. Bars =50μm.

**Figure 4:**
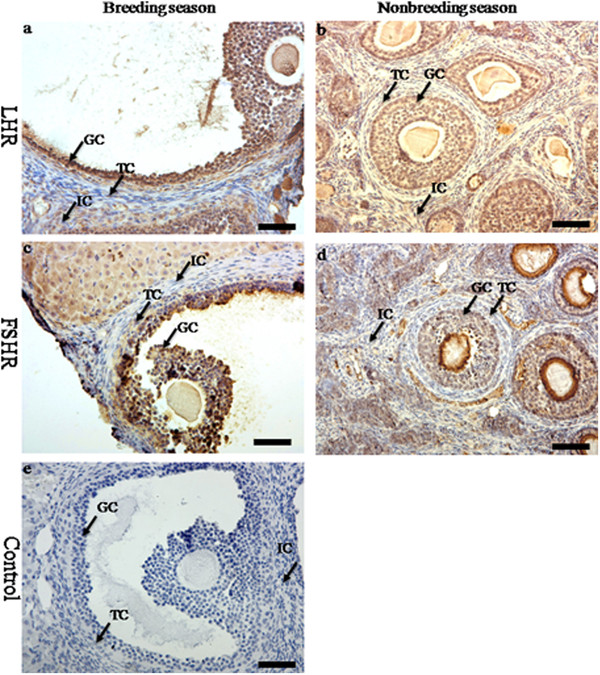
**The immunolocalization of FSHR and LHR in the ovary of wild female ground squirrels.** The immunolocalization of FSHR (**a**, **b**) and LHR (**c**, **d**) in the ovary of wild female ground squirrels during the breeding and nonbreediang seasons. **e**, negative control. IC, interstitial cell; TC, theca cell; GC, granulosa cell. Bars =50μm.

Immunoreactivities of steroidogenic enzymes (P450c17 and P450arom) were detected in the ovaries during the breeding and nonbreeding seasons (Figure 2). The positive staining of P450c17 was localized in the theca cells in the ovaries of the breeding and nonbreeding seasons (Figure 2a, b, c). Meanwhile, immunostaining of P450arom was detected in granulosa cells only in the ovaries of the breeding season (Figure 2)d and e. No immunostaining was detected in control sections when normal rabbit serum was substituted for the primary antibody (Figure 2g). The immunolocalization of AR was observed in theca cells, granulosa cells and interstitial cells in the ovaries of both the breeding and nonbreeding seasons (Figure 3a, b and c). However, seasonal variance in the immunolocalization of ERa and ERb was very similar: they were both present in granulosa cells, theca cells and interstitial cells in the ovaries of the breeding (Figure 3d, e, g and h) and nonbreeding seasons (Figure 3f and i). Negative controls did not exhibit any staining (Figure 3j). The positive stains of FSHR and LHR were found in the granulosa cells and theca cells in the ovaries of both the breeding and nonbreeding seasons (Figure 4a, b, c and d). No immunostaining was detected in negative control sections when normal rabbit serum was used instead of the primary antibody (Figure 4e).

### Western blotting

**Figure 5:**
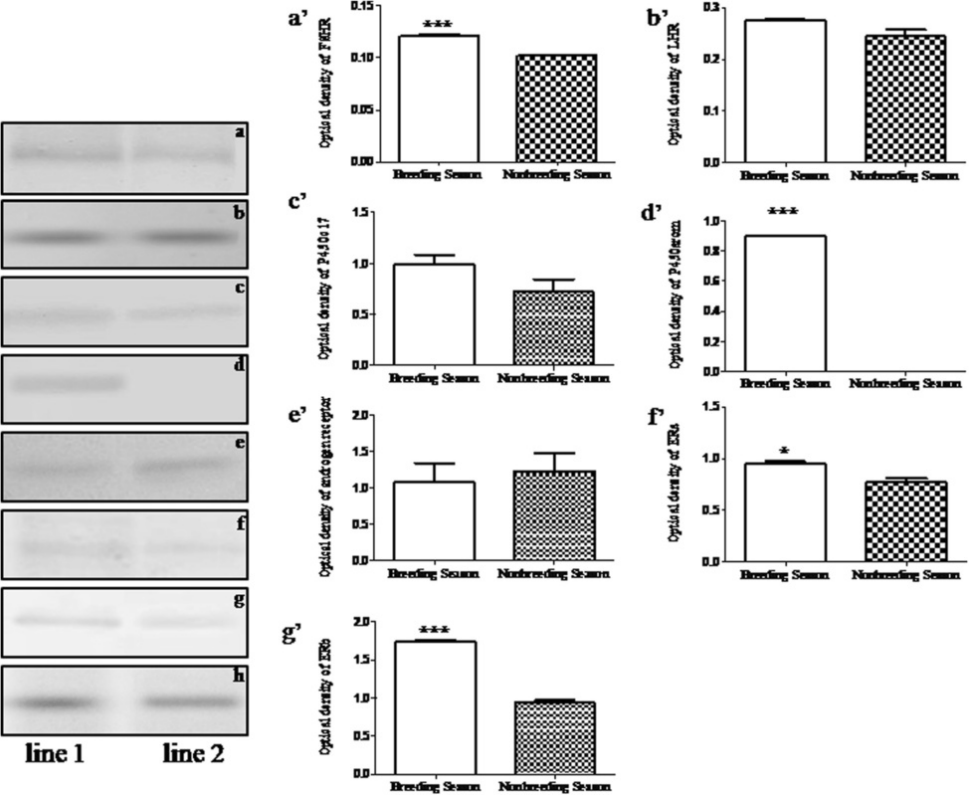
**Western blotting of FSHR, LHR, P450c17, P450arom, AR and ERs in the ovary of wild ground squirrels.** The expression levels of FSHR (**a**), LHR (**b**), P450c17 (**c**), P450arom (**d**), AR (**e**), ERa (**f**) and ERb (**g**) were analyzed by optical density in the ovary of wild ground squirrels. The proteins were extracted from the whole ovary of the breeding season (lane 1), nonbreeding season (lane 2). b-actin (**h**) was used for endogenous control. The analysis of optical density of FSHR, LHR, P450c17, P450arom, AR, ERa and ERb were shown in histogram **a**’-**g**’, respectively. Data are shown as the mean ± SEM. *: p < 0.05, **: p < 0.01, ***: p < 0.001.

**Figure 6:**
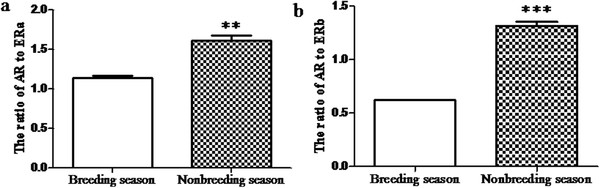
**The ratio of AR to ERs in the ovary of wild ground squirrels.** The ratio of AR to ERa (**a**) and ERb (**b**) in the ovary of wild ground squirrels according to the expression level of AR and ERs. Data are shown as the mean ± SEM. *: p < 0.05, **: p < 0.01, ***: p < 0.001.

The results of Western blotting analysis for FSHR, LHR, P450c17, P450arom, AR and ERs in the ovaries of the breeding and nonbreeding seasons were shown in Figure 5. The proteins extract from the ovaries of the breeding and nonbreeding seasons were loaded in the lane 1 and lane 2, respectively. In addition, b-actin was used as the endogenous control. Meanwhile, the expression levels of FSHR, LHR, P450c17, P450arom, AR, ERa and ERb were analyzed according to the optical density, which were shown in Figure 5 a’-g’ respectively. There was no significant change for the expression of P450c17 between the breeding and nonbreeding seasons (Figure 5c’). However, the expression of P450arom reduced significantly from the breeding to nonbreeding season (Figure 5d’). Similar to the expression pattern of P450c17, the immunoreactivity of AR was not significantly different in the breeding season versus the nonbreeding season (Figure 5e’). Moreover, the immunoreactivities of ERa and ERb were both remarkably reduced from the breeding to nonbreeding season (Figure 5 f’ and g’, respectively). Meanwhile, the immunoreactivities of FSHR and LHR decreased observably in the ovaries of the nonbreeding season when compared with the immunoreactivities of FSHR and LHR in the ovaries of the breeding season (Figure 5 a’ and b’, respectively). In addition, the ratio of AR to ERs was shown in Figure 6. Both the ratio of AR to ERa and the ratio of AR to ERb were increased significantly from the breeding to nonbreeding season in the ovaries of wild female ground squirrels.

## Discussion

This was the first study to investigate the immunoreactivities of FSHR, LHR, P450c17, P450arom, AR and ERs in the ovaries of wild ground squirrels. The results of this study demonstrated that there were primary, secondary, antral and dominant follicles in the ovaries of the breeding season, but only preantral follicles existed in the ovaries of the nonbreeding season in wild female ground squirrels. The protein levels of FSHR, LHR, P450arom and ERs reduced significantly in the nonbreeding season compared to the breeding season. However, the protein levels of P450c17 and AR changed only slightly between the breeding and nonbreeding seasons. These findings suggested that androgen might play an important regulatory role during the follicular development in the ovary of wild female ground squirrels in the transition from the breeding season to nonbreeding season.

In this study, the results of histological observations showed that there were primary, secondary, antral, and dominant follicles in the ovaries of the breeding season, but only preantral follicles existed in the ovaries of the nonbreeding season. This suggested that preantral follicles were unable to continue their development into post-antral follicles, or even develop into corpus luteum in the ovaries of wild female ground squirrels during the nonbreeding season. Similar results were found in other animals, such as domestic sows [24] and buffalos [25]. Porcine oocytes of domestic sows failed to reach their full developmental potential during the period of seasonal infertility. In the buffalos, reproductive efficiency was the primary factor affecting productivity and was hampered in female buffalo by poor estrus expression in summer and distinct seasonal reproductive patterns. These data demonstrated that differences of follicular development existed in the ovaries of seasonally reproductive animals. Follicular growth and steroidogenesis were dependent on the coordinated actions of FSH and LH with their receptors on granulosa cells and thecal cells of ovarian follicles [26]. Previous reports in cattle proved that the follicular wave was associated with elevated expression of FSHR and LHR, and regression of subordinate follicles might be associated with reduction of mRNA expression for LHR and FSHR, suggesting follicular development was closely related to the function of FSH and LH in special follicular stages [27,28]. The present results showed that the immunoreactivities of FSHR and LHR decreased significantly in the ovaries of the nonbreeding season suggesting that follicular development and the immunoreactivities of FSHR and LHR had a positive correlation during seasonal changes in the ovary of wild female ground squirrels.

Generally, ovarian estrogens were believed to regulate follicular maturation locally in the ovary and to stimulate the proliferation of granulosa cells during follicular growth of the dominant follicles [29]. However, the function of androgen in follicular development had received more attention in recent years [30,31]. As two important steroid synthesis enzymes, P450c17 and P450arom had been proven to mediate steroid generation to regulate the follicular growth under the control of LH and FSH, respectively. In our study, P450arom was negative in the ovaries of wild female ground squirrels during the nonbreeding season, indicating that a little amount of androgen was converted into estrogen during this period. This phenomenon was not unique in the ovary of wild female ground squirrels. Similar results had been found in other animals, such as hokkaido brown bears [32] and human polycystic ovary syndrome (PCOS) [33]. In the hokkaido brown bears, the immunoreactivity of P450arom was detected in neither theca cells nor granulosa cells in medium follicles during the mating season, indicating that androgen was unable to convert into estrogen and might play a regulatory role in this follicular stage. In human PCOS, no P450arom expression was detected in granulosa cells of the ovarian follicles, suggesting that androgen might have played a role in the abnormal follicular development. In the present immunohistochemical study, P450c17 was positive in theca cells in the ovaries of both the breeding and nonbreeding seasons. Compared with the immunoreactivity of P450c17 in different stages, no obvious reduction was found comparing the breeding season with the nonbreeding season, which suggested that androgen was synthesized exactly in these two periods. Combining the results of histological observation and the expression level of AR in our present study, these results showed that there were not post-antral follicles in the ovaries of the nonbreeding season and the expression level of AR was higher during the nonbreeding season compared to the breeding season. Previous reviews elaborated that androgen action did play a role in regulating follicle development and ovulation, and that AR-mediated actions might underlie new facets of the hormonal regulation of female fertility, including that androgen could mediate their actions to regulate follicular atresia directly via the AR [15]. Taken together, it appeared that androgen might play an important role directly, via AR, in follicular development during the nonbreeding season in wild female ground squirrels.

Previous studies suggested that androgenic actions played an important role in follicle initiation and early growth [34,35]. Interestingly, the present study showed that there was no distinguishing change in the protein level of AR, however, the protein level of both ERs decreased markedly in the nonbreeding season compared to the breeding season. In the present study, there were no post-antral follicles in the ovaries during the nonbreeding season; it appeared that androgen inhibited follicular growth during the ovaries of the nonbreeding season. Similar results had been reported in mice and humans [15,34]. The late antral and atretic follicles in ERb knockout mice were characterized by a high level of expression of AR indicating androgen might inhibit antral growth via high level of expression of AR [34]. The absence of ER and aromatase expression in the granulosa cells of PCOS might be important in abnormal follicular development in patients with PCOS, suggesting that the absence of ER promoted abnormal follicular development and atretic follicles [15]. During follicular growth, changes in follicular fluid steroid levels had been correlated with follicle health and stage of development [36-38]. In humans, atretic follicles of all sizes observed exhibited an androgenic pattern of steroids in their follicular fluid [38]. According to these studies, the expression level of AR might be higher relatively in atretic follicles. In the present study, it was found in wild female ground squirrels that the AR: ER ratio in the ovaries of the nonbreeding season exceeded the AR: ER ratio in the ovaries of the breeding season. Further studies were needed to investigate the circulating levels of androgen and estrogen in wild female ground squirrels during the breeding and nonbreeding seasons. Taken together, the present study suggested that androgen might mainly convert into estrogen to promote the preantral follicular development during the breeding season, but inhibit the follicular growth during the nonbreeding season in the ovary of wild female ground squirrels.

## Conclusion

In conclusion, wild female ground squirrels might offer a useful animal model to study the regulation pathway of androgen during the process of follicular development in the ovary. Androgen might mainly convert into estrogen to regulate the follicular development via binding estrogen receptors during the breeding season, whereas androgen might bind androgen receptors directly to regulate the follicular development during the nonbreeding season in the ovary of the wild female ground squirrels.

## Competing interests

The authors declare that they have no competing interests.

## Authors’ contributions

XL participated in performing the experiments, analyzing the data and drafting the manuscript. HZ, XS, BL and JZ assisted with sample collection, all experiments and helped revising the manuscript. MX and QW designed and supervised the study, and participated manuscript revision. GW and KT revised the manuscript. All authors read and approved the final manuscript.
